# Transplantation of composite tissue allografts. Scientific bases and clinical applications

**Published:** 2013-03-25

**Authors:** C Giuglea, C Coman, S Marinescu, I Florescu, I Lascar

**Affiliations:** * Clinical Department of Plastic and Reconstructive Surgery, “Sf. Ioan" Clinical Emergency Hospital, Bucharest; ** Clinical Department of Plastic and Reconstructive Surgery, “Bagdasar-Arseni" Clinical Emergency Hospital, Bucharest; *** Clinical Department of Plastic and Reconstructive Surgery, Clinical Emergency Hospital, Bucharest

**Keywords:** allotransplantation, composite tissue allografts, hand transplantation, face transplantation

## Abstract

Plastic surgery is by excellence a surgery in permanent search for new reconstructive options. In the last 50 years researches in the field of allotransplantation led to obtaining promising results for solving difficult cases when autologous tissues are not available for reconstruction, despite all the bioethical issues of this subject. This field of composite tissue transplantation evolved constantly, the top of it being total face transplantation, successfully accomplished while being based on the knowledge accumulated. There are many clinical applications of CTA, hand transplantation or only flexor tendon apparatus, lower limb, partial or complete face transplantations represent the most important part for us, plastic surgeons for obtaining the best reconstruction possible.

## Background

In the beginning of this paper, it would be good to remember the words of Stuart Brand “Once a new technology rolls over you, if you are not a part of the steamroller, you are part of the road". Therefore, allogenic transplantation became a clinical reality only four decades ago. The first successful kidney transplant was performed in 1954 and after that, thousands of transplants have been performed worldwide: transplants of skin, bone and cartilage, cornea, endocrine glands, blood vessels and the most impressive composite tissue allografts. It was a field that grew up fast, involving many disciplines like genetics, tissue engineering, oncology and immunology [**1**].

After the success of transplantation, we had to deal with a new definition of death, meaning loss of brain function and also implied the ethical matters in relation to the use of living donors. Today, we accept that the best summation of the current status of CTA transplantation is a report from June 2006 from “American Journal of Transplantation" entitled: Bilateral hand transplantation – six years after the first case, representing the advancement in microsurgery with regeneration of nerves and muscles of the hand and complete healing of allogenic skin.

 We can define composite tissue allotransplantation as transplanting a graft composed of a variety of heterogeneous antigenic tissues across a genetic mismatch. It is a challenge with many barriers and complexities in comparison to a more homogenous organ such liver or kidney.

 Starting with the year 348 A.D., from which we have the first writing about a CTA, when two brothers from Arabia – Saints Cosmas and Damian – posthumously transplanted an Ethiopian Moor’s limb in place of an elder’s amputated gangrenous limb.

## Current status

Plastic surgery, as we all know, has to deal with severe tissue loss due to trauma, tumors or burns and when has limited options for reconstruction with the autologous tissues can use CTA as a new treatment.

 CTA became a mixture between microsurgical techniques and immunosuppressive agents for the prevention of rejection.

 Until now, we can report from the International Registry of Hand and Composite Tissue Allotransplantation, 74 upper limb transplantations performed until May 2011 worldwide, with a total of 98 CTA transplants [**2**]. 

 In the last 50 years, surgeons have tried to transplant various composite tissues, such as tracheal allografts in 1979, flexor tendon apparatus in 1967, nerve allografts in 1988, knee in 1996, partial face allografts in 2005. We are really looking to the future of total face transplantation which became a reality not long ago [**3,4**].

 In 1967, Peacock reported for the first time a successfully human composite flexor tendon allotransplantation. In fact, between 1957 and 1967, 11 cadaveric transplants were performed for ten patients with no immunosuppression [**5,6**]. The first successfully vascularized flexor tendon apparatus transplantation was reported by Guimberteau in 1988 [**7,8**].

 The first lower extremity transplantation was reported in 2006 with a 3 months follow-up that noted “encouraging return of muscle function with intact neurological sensation". Because the limb was transplanted from the twin sister there was no need for immunosuppression [**9**].

 A major step in composite tissue allotransplantation was made in 2005, November 27, when a team of French surgeons led by Duvauchelle and Dubernard transplanted a triangular allograft containing a distal nose, lips and chin [**10**]. The second partial face transplantation was performed in China in April 2006; it was a reconstruction after a bear bite that contained cheek, upper lip, nose and an eyebrow [**11**].

 As it was expected, the most controversial CTA subject is full-face transplantation. Plastic surgery can do a lot by using classic methods of reconstruction, face area being the most challenging. We have tried many operations beginning with local flaps, expanders and finishing with free prefabricated tissue transfers in order to obtain the same match of color and texture of the lost facial tissues. But, the aesthetic and functional results were pretty much disappointing due to the fact that we need about 1200 cm2 of skin to cover the whole face, scalp, neck and ears [**12,13**]. There are hundreds of people all over the world disfigured after severe trauma, extensive tumors or burns and for them the only option remains full face transplantation. It even seems appealing that this transplant comes together with many psychological and ethical issues [**14**].

## Clinical applications

Practically, CTA can be used in any field of plastic surgery, starting with face reconstruction and ending with lower extremity. With the proper immunosuppression and a very specialized operating team, we can replace almost any missing part of our body.

 We will discuss about the main implications of CTA for plastic surgery, data about each transplant, results and future expectations. The gold principles of every procedure remain the same: the proper selection of the patient who will undergo this transplant, CTA well adapted for the patient’s needs, correct immunosuppression and postoperative care. These goals can be achieved if we have a highly specialized center with a highly specialized surgical team and a standard protocol.

## Lower extremity

There is no question that the largest debate on CTA is on the upper extremity, but progress is made also for the lower extremity. The history of CTA in lower limb reconstruction began in 1270 when Jacopo da Varagine painted “The legend of the black leg" about the two Saints Cosmas and Damian. The true step in lower extremity reconstruction using CTA was made in 1908 when Erich Lexer performed the first composite knee transplant [**15**]. Chiron et al. performed the first vascularized allogeneic transplant of a human femoral diaphysis in 1990 but with no proper immunosuppression the transplant failed [**16**]. Nowadays, this problem was solved and the chances of rejecting the allograft are minimal. Reeves performed vascularized transplantation of CTA on lower extremity on animal models, for more than 30 years, proving their feasibility [**17**]. The first successful vascularized transplantation of a human knee joint was achieved by Hofmann et al., the case being published in 1997 [**18**].

 We believe that when the immunosuppressive therapy will be perfectly established, CTA can become a common practice for reconstruction of large defects of the lower extremity [**1**]. 

## Hand transplantation

The loss of a hand or both is an important functional impediment for the patient; that is why when the possibility of replantation is gone; the only solution remains hand transplantation, with all psychological and immunological problems [**19**]. Since 1998, when the first hand transplantation took place in Lyon, France multiple hand transplants were performed all over the world [**20,21**]. There were two peaks of incidence, with seven hand transplants in 2000 and 2010 with a total of 74 transplants until May 2011 [**2**]. 

 Therefore, as the number of these procedures increased, we gained more and more information and also courage. Due to the fact that immunosuppressive therapy was more effective and rejection problems less present, this procedure became more secure and the results more predictable. Pitsburg protocol of immunosuppression which included monotherapy with tacrolimus or sirolimus and also bone marrow infusion from the donor just after the operation, seems to be safe [**22**].

 Why should we do this procedure? We must have as a main goal the obtaining of a motor and sensor function that enables the patient to regain his motor activities and tactile sensation for quick social reintegration. The outcome after hand transplantation varies from loss of the transplanted hand to 65-70% function of that of a normal hand, better than many hand replantations. The aspects that should be taken into account are: patient psychological stability and capacity of understanding this type of procedure, permanent information of the patient about everything involving the procedure and follow up, a precise protocol and surgical training, clinical implications in both hand amputation, standard immunosuppressive treatment, interdisciplinary approach with rehabilitation team. Still, the number of patients with hand transplant is low but the number will grow in the next period for sure, their number being registered in an International Registry for outcome improvement.

 We can conclude that hand transplantation can be successfully performed with acceptable risk and for selected patients. Although for forearm transplantation the conclusion needs to be studied more, the studies we have undergone until now, suggest that the muscle reinnervation and reactivation is more complex than hand transplantation [**1**]. 

## Face transplantation

Plastic surgery always tried to overcome the limits of traditional reconstruction to obtain the best result in most difficult cases. We have got better and better by using tissue transfer in obtaining very good results for difficult cases, but mutilating lesions of the face remained unsolved [**23**]. That is why the advances in CTA and the latest researches led to partial and complete face transplantation. Before that happened, it took about 15 years of experimental facial transplantation with varying immunosuppressive protocols, as Maria Siemionow describes in her publications. In the last 9 years, face transplantation in humans has been prepared by studying facial skin transplant models in rats to induce tolerance [**24**]. Therefore, it was possible to introduce a full facial or scalp transplant model that crosses all histocompatibility barriers to confirm the feasibility of the total facial or scalp allograft transplantation [**25**]. 

 Many cadaver dissections were performed for the preparation of face transplantation after injecting a contrast substance that enabled us to see the entire vascular territories. The measurement of the skin area showed a surface of 1192+/-38,2 cm with the scalp [**12**]. 

 Mock facial transplantations were also practiced to simulate clinical circumstances on cadavers. They took facial or scalp flaps from the donor cadavers and transplanted them to recipient cadavers with the measurement of the time needed for flaps harvesting and the length of the nerves and vessels included in the flap.

 The sequence of the procedure is as it follows: transfer of the donor facial flap into the recipient’s facial defect, coaptation of the supraorbital, infraorbital and mental nerves, anchoring of the flap at the region of the mandibular and zygomatic ligaments, and also to the preauricular region, mastoid fascia, and temporal fascia, frontal bones, closure of the gingivobucal incisions, closure of the conjunctival incisions, anastomoses of the external carotid arteries between the donor and recipient, anastomoses of the facial veins, coaptation of the great auricular nerves, anastomoses of the external jugular veins and closure of the skin incisions [**1**].

 The studies performed on cadavers proved the feasibility of the face transplantation from the technical point of view and opened the field of “identity transfer" and the debate on ethical, social and psychological issues [**26,14**].

 After 10 months of debate on face transplantation, the Cleveland Clinic Foundation’s Institutional Review Board approved the protocol of Maria Siemionow, allowing the Clinic to select the patients with severe disfigurements as potential candidates for this operation [**27**]. We must look at the future with hope even though we cannot predict the appearance of the recipient’s face. We have computer based modeling that tells us about this aspect, suggesting that the face will take more of the skeleton of the recipient than the soft tissues of the donor.

 As Maria Siemionow said, we have at this time the anatomic science, microsurgical skills and immunological expertise to make face transplantation a clinical reality [**1**].

 Based on all the knowledge we have had until now, in the recent years, we have began studying this procedure, by performing cadaver dissections to simulate the surgical technique. We injected colored latex in the carotid artery to visualize the vascular territories and afterwards we dissected a mock facial transplant, partially and totally. At this moment, we are looking to the future with the hope that when bioethical issues will be solved, imunosuppression therapy protocols established, we will have the proper conditions to deal with extremely difficult cases that have no other surgical option.

**Fig. 1:**
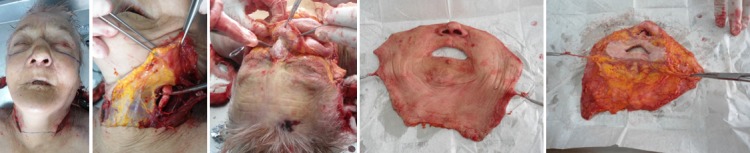
a-e Images with face dissection and harvesting

## Conclusions

Always looking for the best solution to repair what has been damaged, led plastic surgery to the field of composite tissue allotransplantation. All the research from the last fifty years, lab tests, cadaver dissections, immunosuppressive therapy evolution was meant to prove that CTA is a clinical reality. Nowadays, especially hand and face transplantation represent the only solution for desperate cases with no other reconstructive option. We are responsible for the way the future will look like, if the debate on ethical and psychological problems will lead us to the day when these procedures will be as common as the other reconstructive interventions.

**Acknowledgement: **This paper is partly supported by the Sectorial Operational Programme Human Resources Development (SOPHRD), financed from the European Social Fund and by the Romanian Government under the contract number POSDRU 64153
